# Pulse and Entrainment to Non-Isochronous Auditory Stimuli: The Case of North Indian Alap

**DOI:** 10.1371/journal.pone.0123247

**Published:** 2015-04-07

**Authors:** Udo Will, Martin Clayton, Ira Wertheim, Laura Leante, Eric Berg

**Affiliations:** 1 Cognitive Ethnomusicology, SoM, The Ohio State University, Columbus, Ohio, United States of America; 2 Music Department, Durham University, Durham, United Kingdom; Max Planck Institute for Human Cognitive and Brain Sciences, GERMANY

## Abstract

Pulse is often understood as a feature of a (quasi-) isochronous event sequence that is picked up by an entrained subject. However, entrainment does not only occur between quasi-periodic rhythms. This paper demonstrates the expression of pulse by subjects listening to non-periodic musical stimuli and investigates the processes behind this behaviour. The stimuli are extracts from the introductory sections of North Indian (Hindustani) classical music performances (alap, jor and jhala). The first of three experiments demonstrates regular motor responses to both irregular alap and more regular jor sections: responses to alap appear related to individual spontaneous tempi, while for jor they relate to the stimulus event rate. A second experiment investigated whether subjects respond to average periodicities of the alap section, and whether their responses show phase alignment to the musical events. In the third experiment we investigated responses to a broader sample of performances, testing their relationship to spontaneous tempo, and the effect of prior experience with this music. Our results suggest an entrainment model in which pulse is understood as the experience of one’s internal periodicity: it is not necessarily linked to temporally regular, structured sensory input streams; it can arise spontaneously through the performance of repetitive motor actions, or on exposure to event sequences with rather irregular temporal structures. Greater regularity in the external event sequence leads to entrainment between motor responses and stimulus sequence, modifying subjects’ internal periodicities in such a way that they are either identical or harmonically related to each other. This can be considered as the basis for shared (rhythmic) experience and may be an important process supporting ‘social’ effects of temporally regular music.

## Introduction

Pulse is often understood as the subjective experience of an isochronous or quasi isochronous series of sensory events [1]. In this view a series of (quasi) isochronous events affords a predictability of upcoming events that is considered central to the evocation of a pulse. Pulse is essentially a feature of a (sufficiently isochronous) event sequence that is picked up by an entrained subject.

A problem with this view is that synchrony seems to depend on a certain degree of isochrony—with larger deviations from isochrony (>15%) synchronization is thought to be impossible [2]. However, research on entrainment in dynamical systems has shown that isochrony or quasi-isochrony is not a prerequisite for synchronization in system interactions: Since Lou Pecora’s work on chaotic transmitters and receivers in communications [3], it is known that non-periodic and even chaotic systems can synchronize and entrain, and both frequency and phase synchronization have been described [4–6]. Synchronization between non-periodic systems occurs due to interactions that can suppress the divergence of their trajectories, depending on conditions such as parameter regions of the systems, coupling strength, and the difference between the drive and response system. Obviously, synchronization does not require periodic, nearly noise-free oscillators; these are but a special case of autonomous oscillators that can entrain and are rarely found in real life processes such as human music-making.

Entrainment theory and the theory of dynamic attending [7–10] in fact offer a more nuanced understanding of pulse as a phenomenon arising from interactions between event sequence and internal rhythms. Entrainment theory posits that two or more independent, autonomous oscillatory processes, if and when they can interact, influence (entrain) each other mutually, and the degree of influence is dependent on the coupling force(s). Two types of rhythms are thought to be at work in listening to music (and perception in general): internal rhythms (within the subject’s body) that interact with and adjust to external rhythms (in environmental stimuli such as musical sound). Humans display a motoric periodicity, their subjective or spontaneous tempo, when asked to perform a repetitive motor task such as tapping without being exposed to an external event series. In the presence of a regular external periodic signal the internal, adaptable oscillators adjust and the periodicity of motor actions attunes to temporal features of the external signal and is perceived as pulse. In the case of a non-isochronic external signal, as we are going to propose here, internal oscillators still seem to guide repetitive motor actions and consequently lead to the experience of a pulse, however weakly this might be influenced by or synchronized with the external signal.

Few studies have, however, explored entrainment in the context of music which listeners report as evoking a weak or intermittent pulse, or even no pulse at all, such as music found in East Asian, Arab-Turkish and South Asian traditions. The unaccompanied melodic sections of a Hindustani (North Indian) classical music recital—alap, jor and jhala, also known collectively as alap—offer a particularly promising research field, since the music apparently moves through transitions from a non-regularly timed alap to a more regularly timed jor and, finally, to a jhala marked by increased speed, dynamics and rhythmic density. Many musicians and musicologists hold that the initial alap phase, whose main purpose is the melodic exposition of a raga, is strictly unpulsed, although this view has been challenged [11]–[12]. The proposed lack of pulse is itself a puzzle: if human beings are predisposed to produce (quasi-) periodic movements and to entrain to periodicities in their environments, as suggested above, then how is it possible to produce musical styles which appear to many to lack pulse? Could there in fact be a regular pulse underlying the production of this music which is difficult for the listener to perceive (a covert pulse)?

A first analysis of tapping responses to Hindustani alap was presented by Berg [13]. He found that when subjects were repeatedly asked to tap to the same alap section they often responded with different tap rates, but the intra-subject average response rates were related in terms of small integer ratios. Such ratios were also found for some of the between-participants response rates, but subjects did not seem to respond to the same average periodicities of the stimulus sequences (i.e. music sections). Berg also found evidence that subjects response rates were influenced by repetitive pattern in the music and certain musical structures such as mukhras, short cadential phrases with more regular rhythms than the other parts of the alap.

With a different approach Clayton [14] explored the tapping behavior of musicians while performing alap—music which, on subsequent playback, they insisted lacked a pulse, despite the fact that they could be clearly observed producing periodic tapping with free hands and fingers while performing. This study demonstrated entrainment between quasi-periodic movements of performers, suggesting that musical performance may be underlain by a subjective periodicity that is not perceived by listeners.

The present study expands these investigations. We are asking how the behaviors produced by our subjects relate to the quasi-periodic musical stimuli. Do they, in fact, manage to extract periodicities embedded in the musical sound? Is there any evidence that tapping or clapping adjusts in phase towards sound events? How does tapping behavior in ‘unpulsed’ alap relate to that in the pulsed jor section that follows? The use of performed music as stimuli offers a range of realistic and behaviorally relevant stimulus conditions. The music examples of the current study are all taken from performances on the sarod, a plucked string instrument with a fretless metal fingerboard. One reason for this selection was that they are examples of performance regarded as purely ‘classical’ and, therefore, follow a specific set of rules. Another reason concerned the analysis of the music. As the sarod is played with a plectrum, most tone attacks have a sharp rising slope allowing for a precise temporal description of the acoustic event series, and for tests of the relationships between various stimulus features and participants’ behavioral responses.

Specifically, in the first experiment we test whether subjects respond with regular tapping to alap and how these responses compare with those to the more regularly timed jor section. The second experiment was designed to investigate whether subjects respond to any average periodicities of the alap and whether their responses show any phase alignments to the music. Building on Berg’s study we predicted that in both experiments listeners’ responses to the alap are influenced by average periodicities of the music, and show intermittent phase alignment. In contrast, for the jor, due to its greater regularity, we expect to see phase synchronization dominating. In the third experiment we investigate how responses to the sections of raga exposition vary in connection with different temporal features of performances, whether spontaneous tempo is a good predictor of participants’ responses for the alap, and how prior experience with the music influences how subjects respond to it.

In our analysis we apply ideas initially developed in studies on noisy quasi-periodic and chaotic processes that required a reformulation of the criterion that defines phase synchronization or phase locking [6], [15]. It is described in a statistical sense as the existence of a preferred value in the distribution of their relative phase: φ_1,2_ = | n*φ_1_—m*φ_2_| ≤ const. For continuous signals the phase can be extracted by either a Hilbert transform or a wavelet transform [16]–[17]. For discrete signals like performance of musical rhythms or tapping responses, each ‘event’ can be considered as the completion of a new cycle and it is possible to apply a modified ‘stroboscopic’ approach [6], [10], [17], [14]: the phase of one oscillator is observed at moments where the other oscillator completes a cycle. Analysis of the phase relationship between the two oscillators makes use of directional, circular statistics [18]: If the two signals are phase synchronized, the relative phase will occupy a small portion of the phase circle and mean phase coherence is high. On the contrary, lack of phase synchronization gives rise to a relative phase that spreads out over the entire unit circle (uniform distribution), and mean phase coherence is very low. Such an approach offers the necessary tools to look into questions of how people respond to non-periodic, non-metered musical stimuli, whether they can feel and perform a pulse with this music and whether their responses are synchronized with the music.

All 30 participants of the current study gave written informed consent before participation. The experiments were approved by the Institutional Review Board of the Ohio State University.

## Experiment 1

In the first experiment we investigated listeners’ motor responses to a section of Ali Akbar Khan’s performance of Rag Marwa. The excerpt was chosen to allow a comparison of the responses to alap and jor, two parts of the opening section in raga performances that have different degrees of temporal regularity (the jor being clearly more regular than the initial alap section). We expected a larger dispersion of the response IOIs for the alap than for the jor because of the greater variability of the event durations in the former section. We also explored the relationship of the individual response rates for the two sections and examined whether they are related to participants’ spontaneous tempi and/or to features of the stimulus event sequence. Finally we quantified the influence of the music stimulus on the tapping responses by analyzing the synchronization between the stimulus and response events, and tested whether and how synchronization to alap and jor are different. If the stimulus and response events were independent we would expect a more or less uniform distribution of the relative phase: the two processes drift freely past each other. In case of an interaction between the two, the sound events of the music would exert an influence on the tapping events and we would expect a deviation from uniformity in the distribution of the relative phase angles.

### Methods

#### Materials

**Fig 1:**
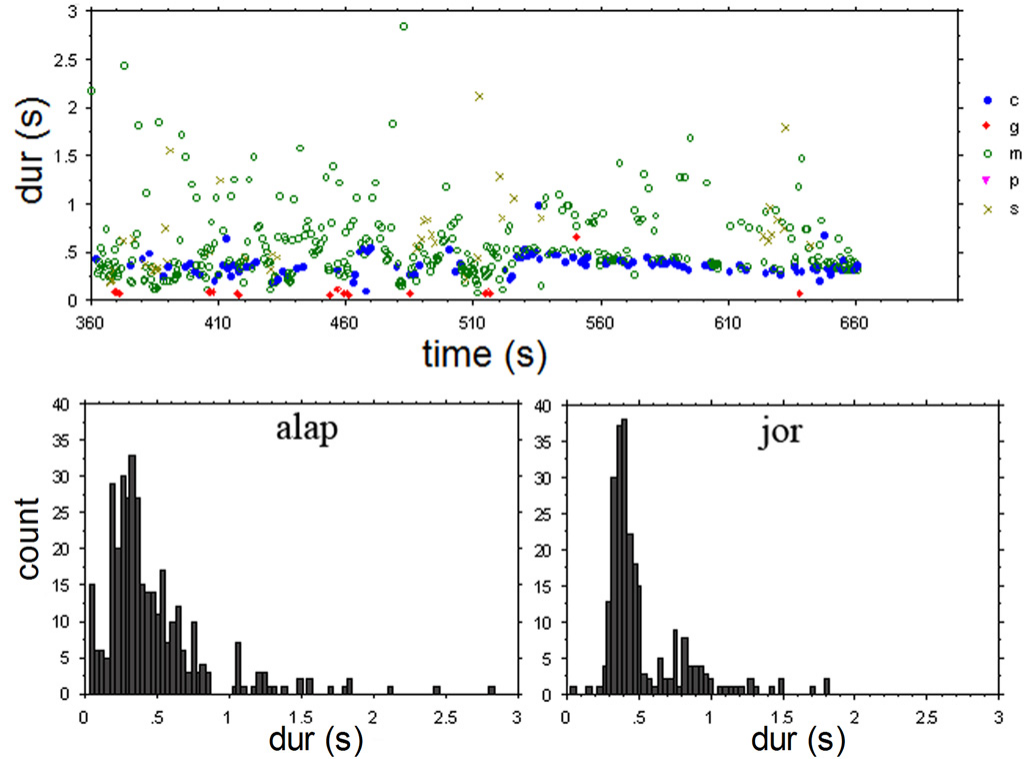
Upper graph: time-duration plot for the Sarod sounds from the excerpt used in Experiment 1. Sound categories: c = chikari (drone strings), g = grace notes, m = main melody (plectrum attack), p = finger picks, s = slides. The jor section, with more regular durations starts at 528 sec. Bottom graphs: event duration histograms for the alap and jor section.

The stimulus used for the experiment consisted of an excerpt from a recording of *Rag Marwa* by Ali Akbar Khan (1994, reissue from the 1970’s). In order to be able to compare responses to irregular and regular pulsed sections we chose an excerpt that comprises minutes 6’ to 11’20” of the performance, consisting of an alap section (from second 360 to 528) and a jor section (from second 528 to 680). The excerpt features the sound of the sarod, which is played—as customary in the North Indian classical music tradition—over the drone provided by a *tanpura* (a plucked long-necked lute). Fig 1 displays the temporal structures of the excerpt, indicating occurrences of events relating to the sarod performance. It is clear from this chart that the punctuating ‘chikari’ strings outline a clear and accelerating pulse in the jor section.

As descriptors for the data dispersion that allow comparison across stimuli and experiments we use below both the coefficient of variation (CV) and the median absolute difference (MAD). The MAD as measure of variability is a robust statistics with good performance for non-normal probability distributions [19]. The respective values (alap (<528 sec): CV = 0.786, MAD = 0.142; jor (≥528 sec): CV = 0.535, MAD = 0.075) clearly indicate the different dispersion for the two sections: the CV for the jor is ca. 2/3 that of the alap, and the MAD is ca. one half.

#### Participants

12 participants took part in this experiment and came from two locations, Columbus, Ohio (4) and Kolkata, India (8). The Columbus subgroup (4 male) consisted of 3 non-Indian graduate ethnomusicology students (one with a degree in sitar performance from Benares) and one Indian sitar player living in Columbus. The Kolkata subgroup consisted entirely of people trained in North Indian Classical music, of whom 7 were professional musicians (5 male and 1 female Indians, and 1 female American), and 1 was a professional Indian sound engineer. Except for two of the Columbus graduates, all participants had extensive performance experience in Indian classical Music.

#### Procedure

After the participants had been seated and introduced to the experimental setup they were first asked to tap (Ohio group) or clap (Kolkata group) for about 1 min at a rate they feel most comfortable with. Participants from the Ohio group were then asked to respond to the music excerpt by tapping with a small metal rod on a non-resonating wooden board. As it was extremely difficult to get the Indian participants to respond in the same way without considerably discomforting them, we modified the procedure for them. With hand clapping being a familiar practice for Indian musicians to indicate units of the rhythmic cycles (talas), we asked them to clap to the music instead of tapping to it. The instruction given was ‘to tap/clap along with the music’. Each participant listened and tapped/clapped to the entire excerpt without interruption. Recording microphones in the two locations (US and India) were adjusted so that taps or claps were recorded on one channel of a stereo recorder, while the other channel received a split line from the music signal that was delivered to the participants via headphones and adjusted to a comfortable volume.

Onset times of both the tap/clap responses and the sound events of the musical excerpt were extracted with an in-house script that determined the maxima of the first derivative of the intensity function of the signals, measured every millisecond with a 10 ms sliding window. The instantaneous phase between responses and stimulus (musical) events was calculated as the location of response events within stimulus event cycles, delineated by the event preceding and the one following the response event [10]. In some instances, especially in the alap sections, there was more than one response event between two stimulus events. In these cases response event selection was done according to the following modification of the Quiroga criteria for discrete event series [17]: Response events must follow or precede a stimulus event; if there are three or more subsequent response events, those not bound by at least one stimulus event were not considered in the analysis, because they are not *directly* influenced by a stimulus event. Resulting time and phase data were analyzed with standard circular statistics packages (R and Oriana). We used Kuiper’s test [20]–[21] to test the null hypothesis of uniform distribution against an unspecified alternative. This avoids making potentially inappropriate a priori assumptions of a unimodal alternative distribution or a pre-specified mean direction, as implied in the Rayleigh test for example. For group comparison we used the non-parametric Mardia-Watson-Wheeler (MWW; [20], [22]).

### Results

**Fig 2:**
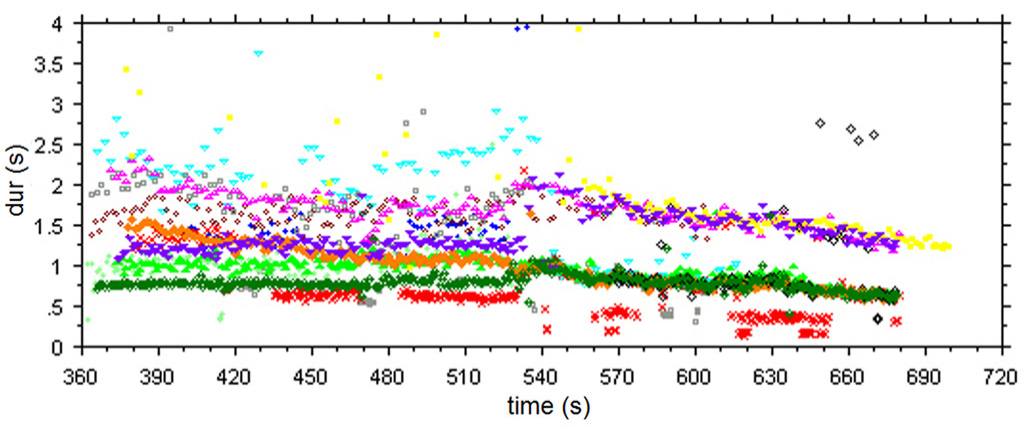
Time-duration plot of tapping responses. Note the unification of response levels during the more regular jor section that starts at second 528.

Most participants responded with regular tapping to both the alap and the jor section. There were two exceptions in the Kolkata subgroup; one musician (s5) clapped along phrase boundaries during the alap section (mean tap duration 4.87 sec), and one (s2) clapped with rhythmic subdivisions and augmentations during the jor section. Although all participants, with the exception of s5, tapped/clapped to the alap section at a relatively steady and regular pace, each one did so by tapping at his or her own rate (Fig 2), with mean durations ranging from 0.778 to 2.181 seconds. A different picture starts to emerge at around second 528 of the performance, the onset of the jor section, where participants’ responses merge into three main ‘streams’ (including s2, who tapped a rhythmic pattern). The main streams are related in terms of ratios of 1:2:4 (considering the midlevel of s2’s responses) and they appear to be transposed versions of each other, including even minor deviations from the overall trend such as around second 540 and 570 (see Fig 2). The time course of the response sequences corresponds to transpositions of the time course of the main event level (marked by the chikari sounds) of this music section by factors 1 (one subjects), 2 (6 subjects), and 4 (4 subjects) (compare Figs 1 and 2).

In order to compare the dispersion of participants’ responses to the two music sections, we first detrended the responses to the jor and then compared the coefficients of variation (CV) for both sections (s5, tapping at phrase boundaries, is not included in the following analysis). There was no significant difference between the two CVs (F (1,10) = 2.992; p = 0.12). In contrast to our initial hypothesis, responses to the alap did not show a larger dispersion than responses to the jor section, even though the dispersion measures (CV and MAD) for the corresponding stimulus sections differ significantly (see Materials section above). There was also no significant correlation between mean tap duration and the CV of the stimulus section (R^2^ = 0.05, adjusted R^2^ = 0.002, p = 0.317).

#### Spontaneous tempo and alap response rate

**Fig 3:**
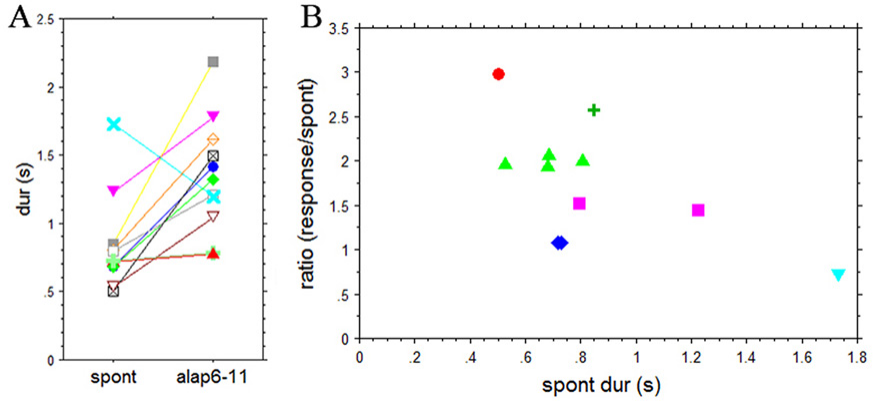
A: Spontaneous tempo (spont) and mean response duration for the alap section (alap). B: Spontaneous tempo (spont dur(s)) plotted against the ratio of response rate and spontaneous tempo. Plot suggests 6 ratio levels: 3:1 (1 subject), 5:2 (1 subject), 2:1 (4 subjects), 3:2 (2 subjects), 1:1 (2 subjects) and 2:3 (1 subject).

Comparing spontaneous tempi and mean responses for the alap we found two subjects showing similar rates for both, one subject increased and the rest considerably decreased response rates for the alap (Fig 3A). Consequently, the two rates show no significant correlation (R = 0.141, R^2^ = 0.02, p = 0.688). However, normalizing the alap response rates to the individual spontaneous rates they form several distinct groups when plotted against the spontaneous tap rates, suggesting a set of distinct ratios between the two rates. For 2 subjects this ratio is close to 1:1, four subjects show a ratio close to 1:2, and for 2 subjects it is ca. 2:3. The remaining 3 subjects show values close to 1:3, 3:2, and 2:5 (Fig 3B). This suggests that spontaneous motor responses and responses to the alap may be related in terms of harmonic ratios. This relationship, however, is not perfect. Overall, the responses to the alap are 1.81% (± 3.89 SD) slower than predicted by the small integer relationship with the spontaneous responses.

#### Phase synchronization

**Fig 4:**
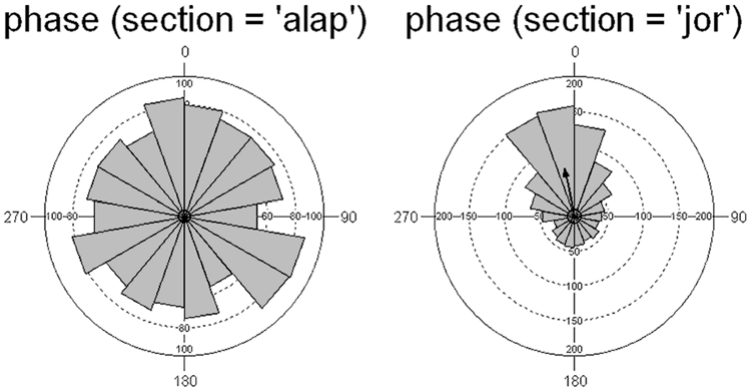
Overall phase histogram for the alap and jor responses.

Analysis of the overall phase alignment between taps and music events shows a rather uniform distribution for the alap (Kuiper V(alap) = 1.118, p>0.15) and a unimodal phase angle distribution with a mean vector orientation of 348° or—12° for the jor, indicating a highly significant synchronization (Kuiper V(jor) = 8.734, p<0.01; Fig 4). The Mardia-Watson-Wheeler test (MWW) shows the differences in response to the two sections to be highly significant: W = 146.691, p<0.0001.

Examining the individual responses for the alap, we find that 3 participants show significant (Kuiper p<0.01) phase synchronization, and for a fourth one the phase alignment approaches significance (Kuiper p<0.06). The phase distribution for these participants shows considerable spread and multimodal peaks for the alignment angles, with alignments both ahead of (e.g. s12 in S1 Fig) and following (e.g. s9 in S1 Fig) music events. For the jor, 8 participants show significant (Kuiper p<0.01) phase synchronization and the phase alignment for two others approaches significance (Kuiper p = 0.055) with more uniform mean angles and dominantly unimodal phase distributions.

### Discussion

In this experiment listeners reacted to both the alap and the jor section with regular sequences of motor responses of comparable variability that did not reflect the different degrees of temporal regularity of the two stimulus sections. Yet, responses to alap and jor differed markedly in the between-subject agreement on response rates. There is little agreement for the alap, with only three cases of two subjects, each showing similar rates. This contrasts with the jor section, where responses are unified at just three different response levels, with the rates at these levels relating to each other in terms of simple integer ratios (1:2:4). While there are at least nine different pulse rates (for 12 subjects) in the alap, there are only three for the jor, and all three are related by simple integer ratios.

These differences may be due to different factors that guide the selection of response levels for alap and jor. Our analysis suggests that spontaneous tempo and mean alap response are related in terms of small integers. This could mean that, rather than being directly related (there was no significant correlation between them in the direct comparison), they could both be related to a common underlying factor like, for example, the resonance period for periodic movements, which in turn shapes spontaneous responses as well as responses to the alap. However, there could be a confounding factor in the current experiment because we combined tapping and clapping responses, even though both of these repetitive motor activities are known to have very similar frequency characteristics in terms of resonance, range and central tendency [23]–[25]. However, an additional test on the K and O sub-group responses did not indicate differences that can be attributed to the different response modes: A Mardia-Watson-Wheeler test on the response phase did not show any sub-group differences for the alap (W = 1.68; p = 0.44) but significant differences for the jor (W = 30.60; p<0.001). If the sub-group differences were due to the response modes they should have shown up in both alap and jor, as the response mode did not change between the sections. Nevertheless, we address this question again in Experiment 3, where we employ a modified experimental design and a uniform response type.

In contrast to the alap, the jor section is characterized by higher temporal regularity, and the basic sound event sequence of this section is formed by chikari and melody sound events of ca. 0.4 sec duration. It seems that this event level acts as a reference for listeners’ responses: one subject tapped at the basic event level of ca. 0.4 sec, 6 subjects selected a level around 0.8 sec, one subject selected 1.2 sec, and 3 subjects selected a level of ca.1.6 sec. Clearly, response rates are strongly shaped by stimulus features. For example, the alternating melody and chikari events—so typical for the jor—produce a prominent timbral-rhythmic pattern with a period of ca. 0.8s, and it is this level that was selected by most participants (6/12) for their response.

Another factor affecting the selection of response levels emerges when we examine the transition between alap and jor (Fig 2). For the jor nine subjects (out of 12) responded with rates that were the same as or close to those of the preceding alap section, and it seems that in the transition to a more regular performance section, listeners have the tendency to stay as close as possible to the response rate selected for the preceding, non-regular section. This suggests that the response level selected during the preceding alap section may strongly influence the level selection for the jor.

There are indications that musical experience might be an additional factor. The slowest and fastest responses were given by two Indian professional musicians. One of them marked phrase boundaries during the alap, but changed to regular responses for the jor. The other musician responded with a mean duration of 0.778 s to the alap and, as the only participant to do so, switched to the basic event level (ca. 0.4 s) for the jor. In both cases it may have been their experience with this kind of music that allowed them to shift their focal attention to levels not attended to by less experienced musicians. The subject giving the fastest responses was a professional tabla player, and his training as well as his expertise in the music enabled him to select an approach to the experimental task that let him attend to this basic event level—an interpretation that finds strong support in his response to the next experiment (see below). Also, the fact that he showed no phase synchronization for the alap but was clearly synchronizing during the jor (see. S1 Fig, bottom row, s2), is probably a consequence of the specific strategy he employed. As he was responding at the level of the average event duration, his responses seem to question the general validity of the suggestion made by Jones and Boltz [26] that the lower stimulus levels do show less temporal predictability. The responses of this musician seem to indicate that predictability may be more shaped by experience and strategy employed than by the selected response levels.

Another difference between responses to alap and jor is the degree of interaction between motor responses and acoustic stimuli or the degree of synchronization between the two. Even though the overall responses to the alap had a rather uniform distribution of phase angles, three subjects showed significant synchronization with the sound events and one subject’s responses approached significance. On the other hand, for the jor section, 83% of the subjects exhibited significant (8/12) or close to significant (2/12) synchronization with the sound events.

Thus, it seems, we can characterize the motor responses to alap and jor as follows. For both sections there appears to be interaction between sound events and motor responses, although, due to its temporal irregularity, this is weak for the alap. The response level (average rate) selection for this section is largely determined by subjects’ internal motor periodicity (as indicated by their spontaneous tempo) and slightly modified by a (weak) interaction with the stimuli. The increased temporal regularity of the jor leads to an attraction of the response levels towards the mean event rate of the stimuli or multiples thereof, and increased synchronization with the event sequence. This entrainment, then, seems to offer a unified experience of pulse across subjects: even if subjects do not tap at the same rate, there are simple common denominators that relate their responses and hence their pulse experience.

## Experiment 2

Here we investigated two follow-up questions arising from the first experiment. Our main question was whether listeners’ motor responses to the alap section, despite the apparent relationship with spontaneous tempo, could be related to statistical features of the stimuli, such as average periodicities, similar to what we found for the jor section. In order to test this, we manipulated an alap section by time stretching to obtain two modified versions, an accelerated and a decelerated one that only differed in duration (speed) of the event sequence. The basic assumption behind this was that if listeners do respond to average periodicities, then the ratio of their motor response rates to the two variants should correspond to the ratio of the duration (or speed) of these variants.

A second question dealt with how representative the results for the first experiment’s alap section were. As alap is a developmental form it is likely that different sections of the alap lead to different degrees of synchronization in listeners’ responses. For the current experiment, we therefore chose a different alap section from the same performance that was used in Experiment 1.

### Methods

#### Materials

**Fig 5:**
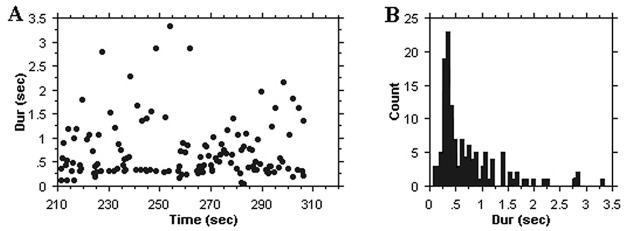
Temporal structure of the sound events from min 3.5–5.1 of the unmodified alap. **A:** time-duration plot. **B:** histogram of event durations. CV = 0.882, median = 0.423, MAD = 0.204.

The stimulus for this experiment was again taken from the *Rag Marwa* recording by Ali Akbar Khan (1994) and consisted of min 3’30” to 5’10” from the alap, a section preceding that used in Experiment 1, and which had a higher CV and MAD (Fig 5) than the alap section from the previous experiment. In order to obtain a reasonable tempo change without distorting the sound we performed two time stretch transformations that produced new versions, one 15% faster and one 15% slower than the original, with no noticeable changes in the sound quality. The change in performance duration from the slow to the fast version was 26.08%.

#### Participants

17 participants formed two groups. The Columbus group (C group) consisted of 9 non-Indian (6 male and 3 female) students from the School of Music, 4 of whom were ethnomusicology students with some listening experience of North Indian music, one being a trained Sitar player. The Kolkata group (K group) was the same as in Experiment 1 and consisted of 8 people, all trained in North Indian classical music. One participant from this group did not give any analyzable responses to the stimuli of this experiment and was excluded from the analysis.

#### Procedure

The procedure was the same as described for Experiment 1, except that participants were asked to listen and tap/clap to two stimuli (the two time-manipulated versions). Half of the participants started with the slowed-down version, and the other half with accelerated version.

### Results

**Fig 6:**
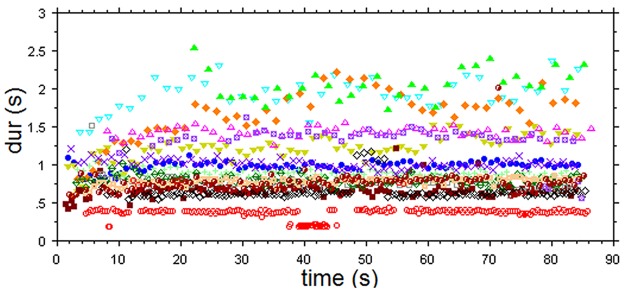
Time-duration plot of tap responses to the fast version of 3’30” to 5’10”of the alap.

As in Experiment 1 (above) and Berg’s study [13], all subjects responded with fairly regular tap/clap sequences to both versions of the alap. There was only one exception, an Indian musician who obviously tapped along with melodic phrase boundaries (with IOIs larger than 3sec). This subject is not considered in the following analyses. Fig 6 shows the responses of the remaining subjects to the fast version.

#### Tapping start time

The presentation order(fast/slow against slow/fast) had no effects on either the start time of the responses or on the mean tap/clap rate (for both: F(1,30)<1). However. the start time was significantly different for the two participant groups (F(1,30) = 23.427; p<0.0001). Mean start times for the slow version were 10.6 s for the K group, and 1.5 s for the C group; for the fast version they were 7.62 s (K group) and 2.01 s (C group). The main effect for group was highly significant (F(1,45) = 22.279; p<0.0001). As both groups were given the same instruction it does not seem likely that these different start times are due to the different response types (taps vs claps) given by the two groups. More likely, the Indian musicians (K group), familiar with the music, take some extra time (average ca. 9 sec) to prescreen the music (for orientation), but when they start their response they do so with a stable rate. This contrasts with the C group subjects, who already start tapping after only1.5–2 sec, but who take longer before they reach a stable level of response.

#### Mean response ratios

In order to compare the individual response rates for the two alap versions we excluded all subdivisions and multiples of the individual mean response levels that were present in some subjects (see Fig 6), as well as the first 20 sec (corresponding to the maximum adjustment period identified for the Kolkata group) from the calculation of the individual means.

**Fig 7:**
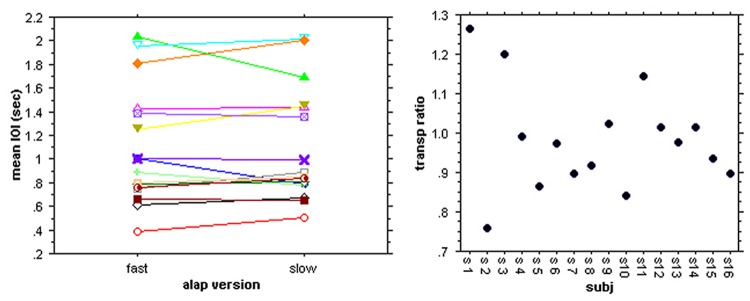
Mean IOI rates for tapping to the slow and fast alap version and their ratios (%) for all subjects.

There were no significant group differences in the change from slow to fast responses. Overall, three subjects maintained the same rate for both versions (ratio of 1 ± 2%), six subjects decreased their rates (ratios >1) and seven followed the direction of the tempo change (ratios <1; Fig 7). Six of the latter show mean IOI ratios from 0.97 to 0.84 and therefore lag behind the actual tempo transposition of the stimulus (0.74). Only the tapping rate change of one subject (0.757; s2 in Fig 7) matches that of the music within 2%, suggesting that his responses are largely determined by average temporal properties of the event sequences.

The individual differences in response to the slow and fast version suggests that, with the exception of s2, subjects attended to different stimulus features in the two presentations.

#### Phase alignment

**Fig 8:**
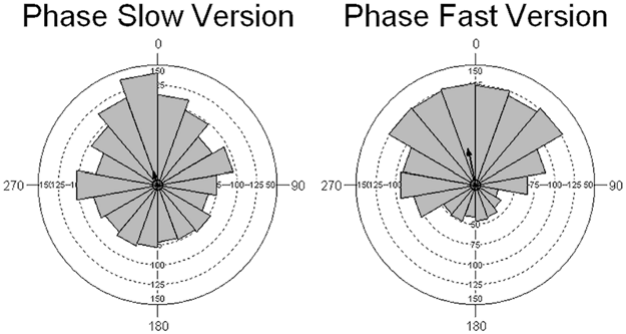
Phase histograms for tapping to the slow and fast version of min 3.5–5.1 of rag Marwa.

The tap phase distributions for both versions of the alap section show highly significant deviation from uniform distribution: Kuiper (slow) V = 3.368, p<0.01, Kuiper (fast) V = 8.296, p<0.01 (Fig 8). The phase distribution for the fast version has a higher concentration (0.637) and a larger mean vector (r = 0.303) than that for the slow one (concentration: 0.24; r = 0.119), however, the angle of the mean vector only differs by 10° (slow: 339°; fast: 348°. The differences between them are highly significant (Mardia-Watson-Wheeler W = 44.958, p<0.001).

The overall responses to the two versions of the music show no significant differences between the Kolkata and the Columbus group. The Mardia-Watson-Wheeler test gives W = 3.271 with p = 0.195 for the slow, and W = 1.036 with p = 0.596 for the fast version.

Inspecting the individual phase distributions we find that 12 participants (out of 17) show significant phase synchronization for both the slow and fast excerpts (e.g. S2 Fig, middle row, S8). Phase alignment for 3 participants is not significant in the slow but significant in the fast version (e.g. S2 Fig, bottom row, S14). This does not appear to be due to a learning, habituation, or presentation sequence effect because two of the three participants responded to the fast version first and the third participant heard the inverse sequence. One participant only approached significance (Kuiper p = 0.058) in the fast version, and one participant did not show significant phase alignment for either version. Interestingly, this participant (S2 Fig, top row, S2) is the only one whose tapping ratios matched the duration shift and the same statistical descriptor (median) for the two stimulus versions, suggesting that this participant followed a different strategy in responding than did most or all others.

### Discussion

The analysis of the response phase distribution shows overall significant synchronization (Kuiper p<0.01) for both stimulus versions. The difference between the two distributions suggests that the faster version leads to slightly more homogeneous responses, and a larger number of significant individual phase alignments. Overall, responses to this 90 second alap excerpt show a higher degree of synchronization than those to the alap section of Experiment 1, and most subjects showed significant phase synchronization to the slow (12/17 subjects) and fast version (15/17 subjects). This suggests that 1) even within one alap performance response synchronization may vary from section to section, and 2) that the degree of synchronization does not seem to correlate with the dispersion of the event intervals: both CV and MAD were larger for the current section (CV = 0.882, MAD = 0.204) than for that of Experiment 1 (CV = 0.786, MAD = 0.142). It is therefore likely that other signal features, such as the recurrence of timbral-rhythmic pattern, have a more significant influence on the degree of synchronization than the dispersion measures. We will return to both these points in the description of Experiment 3 where we discuss whether these results can be generalized to other alap performances, and directly compare phase analysis for alap subsections of different performances.

In this experiment we also examined whether subjects’ responses to alap are shaped by statistical temporal properties of the stimuli. If that were the case we expected the individual response rates to change according to the change in duration of the two stimulus versions. However, our results show that, with one exception, this is not the case. The second stimulus appears to lead to a re-evaluation of stimulus features and re-adjustment of response rates that may be due to a shift in attention towards different features in the second version. The attraction of underlying temporal regularities does not appear strong enough to keep the attentional focus on the same features for both versions.

The one subject whose response ratio for the slow and fast version corresponded to their transformation ratio, thereby indicating mean period alignment, was also the only subject that did not show phase synchronization for either stimulus version. His significant phase synchronization for the jor section of Experiment 1 shows that he is well able to perform phase synchronization but that the listening and tapping strategy he employed for the alap sections lets him attend to a low temporal event level (he has the shortest response period of all subjects) in a way that leads to period synchronization. As mentioned before, this subject was a highly experienced tabla player, and the fact that his response rates changed by the same proportion as the speed changes for the two stimuli suggests that he employed a strategy that permitted him to pick up temporal features of the music not apparent or accessible to any other subject (including other professional Indian musicians). The fact that period synchronization was observed in his case is therefore compatible with the idea that this type of synchronization is cognitively more demanding and more sensitive to attentional requirements than phase synchronization [27]–[28].

## Experiment 3

This experiment addresses the question to what extent the above results depend on the specific temporal structure of the chosen music example. We decided to test excerpts from three different raga performances, played by different musicians, in an experimental design similar to Experiment 1. We selected two excerpts with alap, jor, and jhala sections, and one long alap with three identifiable subsections (the jhala has a faster pace than the jor and its melody notes are interspersed with several plucks on the ‘chikari’ strings tuned to the melody tonic). Response rates for the alap sections were again compared to spontaneous responses to test the generalizability of the results from Experiment 1. The synchronization analysis also addresses the methodological question whether analysis of complete sections is an optimal strategy to detect synchronization in alap. Finally, we readdress the question whether listeners familiar with the music align differently to the music than those unfamiliar with it, following an observation in Experiment 2. In the previous experiment we found significantly different response start times for the participant groups, but there was no indication that they synchronized differently. However, as there were procedural differences between the two groups (one group tapping, the other one clapping, different locations) we decided to replicate this test but with exactly the same conditions for the two groups. Again our hypothesis is that if prior experience has an effect on the way listeners synchronize with the music we should find different circular variance, mean phase angle and mean vector size for the two groups.

### Methods

#### Materials

As stimuli for this experiment we chose excerpts from three different Classical Hindustani sarod performances: the first 9.2 min from Rag Gaud Sarang by Vasant Rai (1975,Vanguard Nomad, SRV-73013), the first 14.8 min from Rag Jaijaivanti played by Budhadev Das Gupta (1993, Nimbus, NI-5134), and the first 13.5 min from Rag Kafi Kanada played by Amjad Ali Khan (2002, Navras, NRCD-0159). The first two excerpts comprised alap, jor and jhala of the performances with the following durations and statistics.

Gaud Sarang: alap 0–4:03:7 (CV 0.748, MAD 0.260), jor 4:03:7–6:41:3 (CV 0.531, MAD 0.059), jhala 6:41:3–9:05:5 (CV 0.669, MAD 0.022). For some analyses the alap was further subdivided (following the mukhra section marker) at 2:17, with subsection alap1 from 0–137 s, and alap2 from 137–243 s.

Jaijaivanti: alap 0–8:04:1 (CV 0.935, MAD 0.123), jor 8:04:1–12:43:5 (CV 0.612, MAD 0.052), jhala 12:43:5–14:50:5 (CV 0.671, MAD 0.176). For some analyses the alap was further subdivided (following the mukhra section marker) at 4:25:1, with subsection alap1 from 0–265 s, and alap2 from 265–484 s.

Kafi Kanada (only alap part of the performance): alap1 0–5:05:38 (CV 0.703, MAD 0.394), alap2 5:05:38–9:47:1 (CV 0.760, MAD 0.214), alap3 9:47:1–13:31:2 (CV 0.658, MAD 0.207).

**Fig 9:**
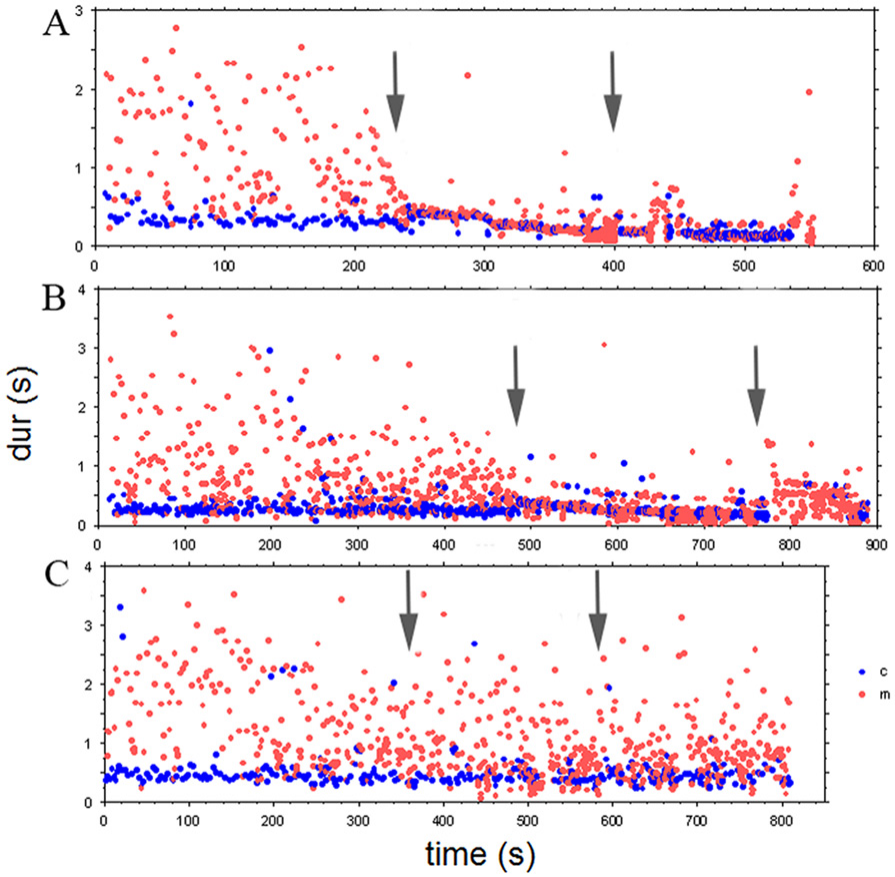
A: time-duration plot of the sound events for the Gaud Sarang excerpt. The jor section starts at 243 sec and the jhala section at 401 sec (marked by arrows). B: time-duration plot for the Jaijaivanti excerpt. The jor section starts at 484 sec and the jhala section at 763 sec (arrows). C: time-duration plot for Kafi Kanada excerpt. Alap section 2 starts at 360 sec and alap section 3 at 587 sec (arrows). Sound category labels: c (blue dots) = chikari (drone strings), m (red dots) = main melody.

Sound events in all three excerpts were classified as chikari (c) or melody (m) sounds. Fig 9 shows the time-duration plots for the three stimuli.

#### Participants

13 participants took part in this experiment. 6 participants were graduate music-major with no training or listening experience in Hindustani music (N group), the other participants were 6 non-music Graduate students of Indian origin and one American music-major graduate, all with training and listening experience in Hindustani music (E group). None of the participants had taken part in the two previous experiments.

#### Procedure

Experimental setup, response recording, data extraction and analysis were the same as in the previous experiments.

After the participants had been seated and introduced to the experimental setup they were first asked to perform a series of claps for 1 min at a rate they feel comfortable with (their spontaneous tempo), then to listen and clap to the three musical examples, and finally to clap again at a comfortable rate for 1 min without musical stimuli. The task sequence for all participants was 1) spontaneous clapping, 2) clapping to the three music excerpts, and 3) again spontaneous clapping. The sequence of the three music excerpts was balanced across the participants, and the instruction given to the participants was simply to ‘clap along with the music’. Following Berg [13] we presented a ‘distractor’ sound file between each clapping task; here we used 25 seconds of pink noise, during which participants did not clap.

### Results

**Fig 10:**
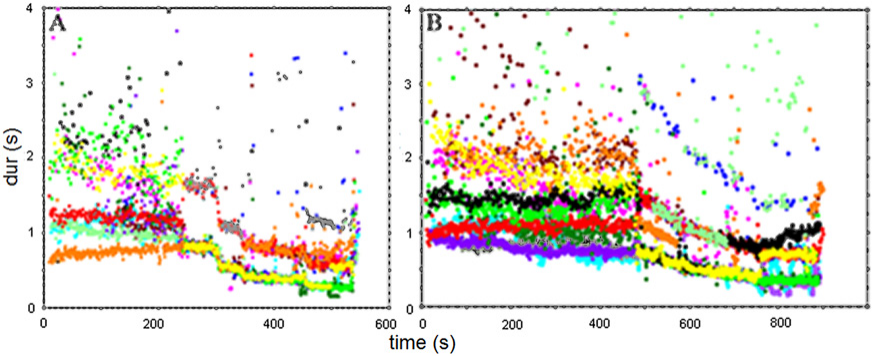
Time-duration plot of the tapping responses to Gaud Sarang (A) and Jaijaivanti (B). Note the changes in response agreement starting with the jor at around 243 sec (Gaud Sarang) and around 484 sec (Jaijaivanti).

**Fig 11:**
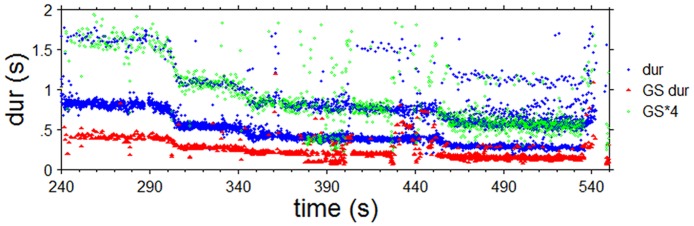
Time-duration plot for the jor and jhala section of Gaud Sarang. Dur (blue) = subject responses, GS dur (red) = music events, GS*4 (green) = music event durations multiplied by 4 to show the overlap with one stream of the subjects’ responses.

As in Experiment 1, participants responded with regular clapping to all alap, jor and jhala sections (Fig 10). The mean rates of their responses to the alap section ranged from 0.742 to 2.690s in Gaud Sarang (GS), 0.831 to 3.133s in Jaijaivanti (JJ) and 0.727 to 3.398s in Kafi Kanada (KK), and there was little agreement across participants. Again, the picture changes with the start of the jor section, where responses merged into two (for GS) and 4 main ‘streams’ (for JJ; see Fig 10B). As was the case in Experiment 1, these main streams are related in terms of small integer ratios to the time course of the main event level (indicated by the regular chikari durations in Fig 9) of these sections: 1:2:4 for GS (see Fig 11 for the match of stimulus event duration multiplied by 4 with the responses at the corresponding level) and 1:2:3:4:8 for JJ, with most participants starting to respond at the 1:2 level for both excerpts (11/13 for GS 8/13 for JJ), followed by the 1:4 level (2/13 for GS, 3/13 for JJ). For Jaijaivanti one subject responded at the 1:8 level throughout the jor/jhala section. Another subject responded first at the 1:3 level but switched to the 1:2 level at ca. 580 s, and to the 1:4 level at ca.630 s. For Gaud Sarang one subject switched to the 1:8 level in the jhala section (Fig 10A). Switches between the 1:2 and the 1:4 level also occurred in other subjects (9/13 for GS and 5/13 for JJ). The fact that these switches occur at different times for different subjects, though dominantly around the transition from the jor to the jhala section, is an additional indication that subjects were attending and responding to different stimulus features.

#### Phase synchronization: Gaud Sarang

**Fig 12:**
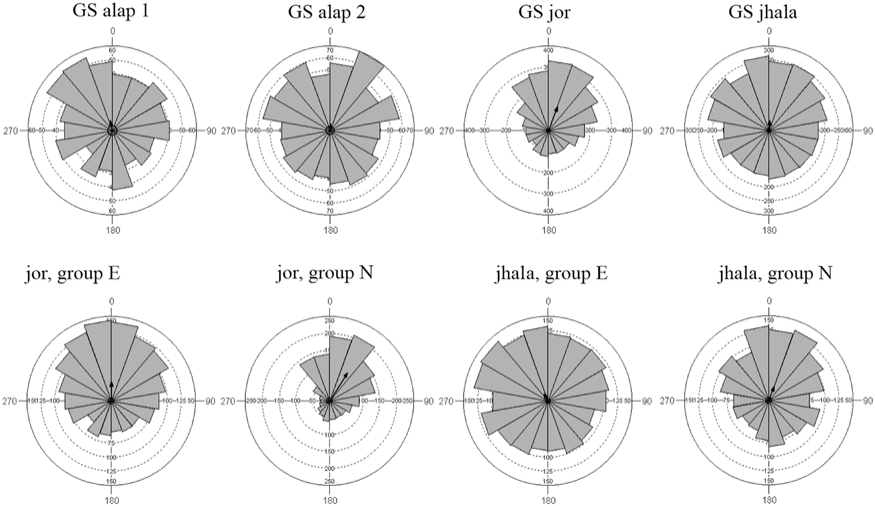
Phase plots of the tapping responses to Gaud Sarang. Top row: all subjects, split by sections. Bottom row: phase plots for jor and jhala section, split by group (E: experienced listeners; N: non-experienced listeners).

Analysis of the overall phase alignment between taps and music event shows that there is synchronization in all four sections (Fig 12, upper row). For alap1 (<137 sec) we obtain Kuiper V = 2.54, p<0.01; for alap2 (137–243 sec) Kuiper V = 2.26, p<0.01; for the jor (243–401 sec) Kuiper V = 11.99, p<0.01; and for the jhala (>401 sec) Kuiper V = 5.08, p<0.01).

If we examine the individual responses (S1A Table) we find that for the first alap section 5 (out of 11) participants show significant (Kuiper p<0.01) phase synchronization. Phase alignment is weaker for the second alap section with 2 participants showing significant alignment and 3 close to significance (Kuiper p = 0.06). Notably only one of the participants shows significant alignment in both alap sections, while the alignment for the other participants is significant only for one section. For the jor the alignment for 11 (out of 13) participants is significant and one close to significance; and 12 (out of 13) show significant synchronization for the jhala section.

In all four sections experienced (E group) and non-experienced (N group) listeners show different length and angles for the mean vector. For alap1 these differences in the phase distribution reach significance (MWW W = 8.13, p<0.017), for alap2 they are not significant (MWW W = 3.86, p<0.15), for the jor (MWW W = 110.76, p<0.0001) and the jhala (MWW W = 30.42, p<0.0001) they are highly significant (Fig 12, bottom row). The vector orientation and length (μ/r) for the E group is 311°/0.07 (alap1), 349°/0.127 (alap2), 359°/0.23 (jor), 333°/0.09 (jhala), and for the N group 1°/0.18 (alap1), 40°/0.045 (alap2), 32°/0.39 (jor), 21°/0.17 (jhala). The overall mean phase angle is 349° (~ -11°) for E and 27° for N group, E having a slightly smaller R and concentration than N (r: 0.14/0.241; c: 0.283/0.497), and the overall differences are significant (MWW W = 116.49, p<0.0001).

#### Phase synchronization: Jaijaivanti

The overall phase alignment between taps and music event turns out to be significant for alap1 (Kuiper V = 3.23, p<0.01), not significant for alap2 (Kuiper V = 1.39, p>0.15), and highly significant for the jor (Kuiper V = 14.68, p<0.01), and the jhala (Kuiper V = 7.48, p<0.01).

For the individual responses (S1B Table) we find that for the first alap section 5 (out of 12) participants show significant (Kuiper p<0.01) phase synchronization. For the second alap section there is only one participant close to significance (Kuiper 0.1>p>0.05). For the jor the alignment for 12 (out of 13) participants is significant and 9 (out of 13) show significant synchronization for the jhala section.

Examining the alignment for experienced (E group) and non-experienced (N group) listeners there are no significant differences for the alap sections (alap1: MWW W = 0.021, p = 0.99; alap2: MWW W = 2.822, p = 0.24). For the jor the two groups show different vector length and concentration (MWW W = 73.24, p<0.0001) and for the jhala these differences are much reduced and not significant (MWW W = 4.92, p<0.085). The mean vector orientations shown by the E and N group are 354° and 347° for alap1, 323° and 42° for alap2, 342° and 350° for the jor, and 3° and 5° for the jhala. The overall mean phase angle is 347° (~ -13°) for the E and 354° (~ -6°) for the N group, with E having slightly smaller r and concentration than N (r: 0.16/0.24; c: 0.324/0.459). Overall difference between the two groups is significant (MWW W = 36.48, p<0.0001).

#### Phase synchronization: Kafi Kanada

**Fig 13:**
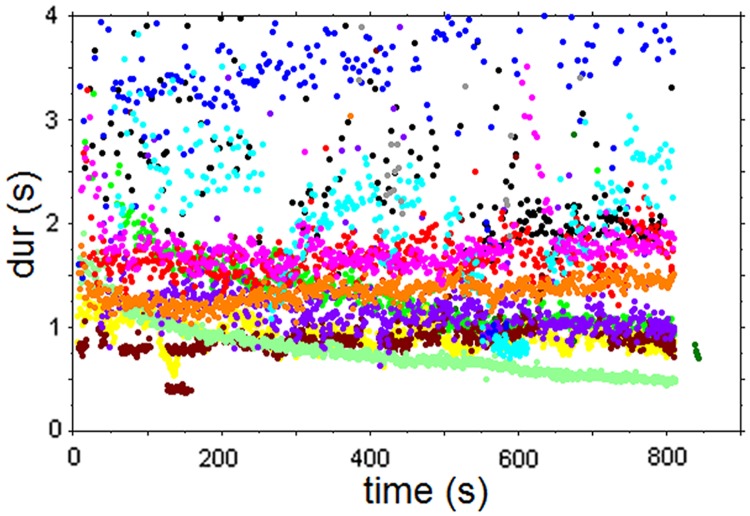
Time-duration plot of the tapping responses to alap of Kafi Kanada, with section2 starting at 360 sec and section 3 at 587 sec.

The overall phase alignment between taps (Fig 13) and music events shows no significant synchronization for alap section1 (Kuiper V = 1.19, p<0.15), a significant synchronization for alap section2 (Kuiper V = 2.55, p<0.01), and again no significant alignment for alap section3 (Kuiper V = 1.37, p<0.15).

Examining the individual responses (S1C Table) we find that for the first alap section 2 (out of 11) participants show significant (Kuiper p<0.01) phase synchronization. For the second alap section 2 (out of 12) participants show significant alignment and 1 is close to significance (Kuiper 0.1>p>0.05), and the same is found for alap section3. Only one of these participants shows significant alignment in all three sections, the other show significance only for one section.

Phase distribution differences for experienced (E group) and non-experienced (N group) is close to significance for alap section 1 (MWW W = 5.179, p = 0.075), but not for alap section 2 (MWW W = 2.142, p = 0.34) and section 3 (MWW W = 3.199, p<0.20). The vector orientations shown by the E and N group respectively are 87° and 337° for alap1, 41° and 63° for alap2, and 67° and 330° for alap3. The overall mean phase angle is 60° for the E and 355° (~ -6°) for the N group, with the overall difference between the two groups only approaching significance (MWW W = 5.965, p<0.058).

#### Synchronization and statistical descriptors

**Fig 14:**
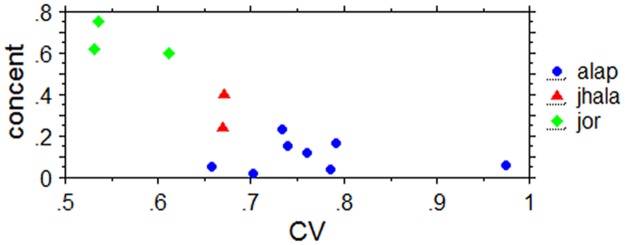
Plot of overall phase angle concentration (concent) of the tap responses against the coeficient of variation (CV) of the stimuli. Data are for the performance sections of the three excerpts from this experiment and the corresponding measures from Experiment 1.

The next question to address concerns the relationship between the degree of synchronization shown by the participants in response to the different performance sections and the corresponding statistical descriptors. Neither CV nor MAD turned out to correlate with either vector lenth or concentration. Rather, a plot of the concentration against CV suggests a kind of step function at around a CV of 0.65 (Fig 14): if the CV is smaller than 0.65 most subjects show synchronization with the stimuli, if CVs are larger than that, no more than half of the subjects show overall synchronization. There is no clear indication that in either of the ranges above or below a CV of 0.65 there is any correlation between the strength of synchronization and CV values. This could suggest that other factors than the selected statistical descriptors influence subjects’ tapping responses and result in significantly different degrees of synchronization in response to different alaps or even alap sections.

#### Spontaneous clapping and response levels in alap

Analyzing participants’ spontaneous clapping we found that, though most subjects clapped at a steady rate, four of our subjets (~31%) switched to a new pace following an initial sequence of spontaneous claps. The new rate was either half (1 subject) or double (3 subjects) the starting rate, and one of the latter subjects switched shortly back to the initial rate towards the end of this task (S3 Fig). We will come back to this revealing result in the dicussion; for the following analysis we only considered the longer, stable part of their spontaneous claps.

**Fig 15:**
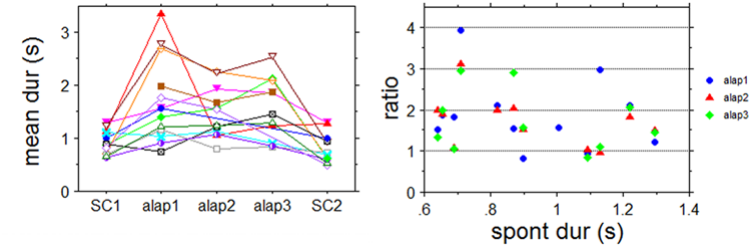
A: Spontaneous clap rates (SC1, SC2) and their relationship to the tap rates for the first, second, and third alap plotted by subject. B: Plot of spontaneous tempo (spont dur(s)) against normalized alap response (ratio) for the alap sections of the three stimuli.

Though there was a significant correlation between the first (SC1) and the second spontaneous clapping responses (SC2) (p = 0.0035), there was only a weak correlation between SC1 and the mean tap rate for the first alap section of each of the three excerpts. For the three correlations r was found to be between 0.33 and 0.38, R^2^ between 0.11 and 0.14, and none reached significance (p between 0.22 and 0.31), indicating that clap rates to alap and spontaneous tempo are not related in a simple 1:1 fashion (Fig 15A). Normalizing the alap response rates to the individual spontaneous rates and plotting them against the duration of the spontaneous claps they seem to form several distinct groups, suggesting a small set of distinct ratios between the two rates, similar to Experiment 1. For all three alap sections together the ratios centered around values of 1:1, 2:3, 1:2, 1:3 and 1:4. While 1:4 only occurs once, 1:1 and 1:2 are the most frequent values with 11 and 10 occurrences, respectively (Fig 15B). Notably, three subjects maintained the same response level (and, therefore, ratio) for all three alap sections, even though these sections were ‘interrupted’ by jor and jhala sections and not continuous. Five subjects showed one level change from the first to the second alap, and the remaining subjects, selected different response levels for each of the alaps. Overall the match between the harmonics of the spontaneous tempo and the alap responses are slightly better than in Experiment 1. The mean deviation from the harmonics was 1.2% ± 9.1(SD) for all three alap sections, with individual deviation of up to 18%. That means, alap response rates are slightly slower than the rates predicted by a small integer relationship with the spontaneous responses.

### Discussion

The main aim of this experiment was to examine to what extent results from the previous two experiments can be generalized to other raga performances. It confirms that subjects produce quite regular responses to alap as well as to the more regular jor/jhala sections, and that the responses differ in their intersubject agreement. Response rates for the alap seem to be related to the individual spontaneous tempi, and therefore show hardly any cross-subject agreement. This is different for the jor/jhala sections, where responses are related to the mean stimulus event period, with notable agreement between subjects, and all response rates related in terms of small integer ratios.

However, our interpretation of Experiment 1 that the response to the first alap section determines the overall response level, with adjustments in subsequent sections, if necessary, following the shortest distance, did not turn out to hold generally, but only seems to be one possible strategy. Responses to Gaud Sarang and Jaijaiwanti show that there can be smooth transitions as well as considerable rate changes in transitions from alap to jor or jor to jhala. Even within the jor and jhala sections we found some subjects changing their response rates due to specific changes in rhythmic patterns that seem to favor responses at certain levels. This is clearly shown for instance in the jhala of Jaijaiwanti, where the performer uses a couple of rhythmically vague playing techniques, such as tremoli, and several subjects change their response level, and consequently show a lesser degree of synchronization than in the more regular jhala section of Gaud Sarang.

#### Synchronization to alap

For all performances we found that alap sections show a smaller degree of phase synchronization than jor and jhala sections, which can be related to the contrast in temporal regularity of these section. Furthermore, we also found performance-dependent variation in synchronization for all alap sections: in the first excerpt synchronization was significant for both alap sections, in the second and third excerpt it was only significant for the first and second section, respectively. This indicates that for the detection of synchronization, especially in alap, the selection of units to be analyzed is relevant, and it seems advisable to either perform a sectioned analyses following musical section markers like the recurrent mukhras, as we have done here, or to perform a windowed analysis, as was for example applied by Patel et al. [29].

The current experiment highlights that alaps from different performances as well as sections from the same alap performance can lead to different degrees of response phase synchronization. Stimulus features that can account for different degrees of synchronizastion, such as the recurrence of temporal features or the overall event speed (as indicated by Experiment 2), are not well captured by common statistical descriptors. Though techniques for the analysis of such features, like visual recurrence analysis [30] and recurrence quantification analysis (e.g. [31]–[32]) have been developed, we found that currently available methods are not sensitive enough to capture the relevant features of our stimuli. A quantitative investigation on how these temporal regularities, or other recurrent structural features such as dynamics or melodic contours, influence synchronization will require the adaptation of recurrence analysis tools to the specific needs of stimuli like the ones used in the present study.

#### Spontaneous tempo and alap response rates

Results concerning the relationship between spontaneous clapping and reponses to the alap are in agreement with results from the first experiment and indicate that they are related in terms of small integer ratios. However, we suggest that this relationship is a reflection of both being related to the subjective or internal periodicity of the human sensorimotor system. This internal periodicity is characterized by a dominant response mode and a set of (harmonically related) minor response modes at multiples of the dominant period. Studies on brainwave coherence response to periodic auditory stimuli [33], and long-term spectra of spontaneous periodic human locomotion [34] suggest that period length of the dominant response mode is about 500ms. For spontaneous responses, i.e. those performed without external stimuli, the majority will be in the dominant mode, but there is a certain likelihood for responses in other, especially neighboring, modes to occur. This would explain the relatively large range of spontaneous motor tempi reported for tapping experiments (e.g. [35], [36], [23]). Further evidence that modes other than the dominant mode of the subjective periodicity can be involved in spontaneous responses comes from the 31% of our subjects that switched their response mode during the spontaneous clapping task (see S3 Fig).

Examining the relation between spontaneous tempo and synchronization tapping to acoustic stimuli with regular perodicities, Drake et al [9] found that listeners are drawn towards longer time spans than their spontaneous tapping period when establishing a referent level. The current experiment indicates that this may also hold for irregular stimuli like our alap excerpts, with 10 out of 12 participants (83%) showing a slower tap rate for the first alap than for the spontaneous tapping. Here, however, the levels participants are drawn to appear to be the (minor) modes of the internal periodicity. Deviations from these modes that were identified, suggest that the actual response rates may be a compromise between the mode of the internal periodicity and salient temporal features of the stimuli. In the case of alap, however, this interaction is relatively weak and the response rate therefore dominated by modes of the subjects’ internal periodicities. This would explain the relative regularity of the tapping responses as well as the diversity of the response rates, each subject having his/her own internal periodicity. This contrasts with responses to jor and jhala sections. The greater regularity of these sections leads to stronger interaction and coupling between internal (responses) and external (music) periodicities, with the responses being drawn and entrained towards the periodicity of the stimuli, resulting in a larger intersubjective agreement on the response rates.

#### Experienced vs non-experienced listeners

Concerning differences between experienced and non-experienced subjects, there were no significant differences between the two groups in terms of the number of subjects that show significant synchronization, but there were significant differences in the way they aligned with the stimuli. Notably, group differences were highly significant in the jor and one of the jhala sections, but significant for only one of the five alap sections. This indicates that effects of listeners’ experience on synchronization measures are larger in responses to regulary timed music than to the irregular alap sections. Perhaps surprisingly, for the more regular jor and jhala sections, the non-experienced group shows a larger concentration for the phase distribution than the experienced group (e.g. Fig 12). However, inspection and comparison of the individual responses shows that this is not due to a weaker phase synhronization in experienced subjects but to larger intersubject variability for this group. Due to different training and experience with this music they seem to employ a variety of listening strategies that lead to different alignment with the music. Responses of the non-experienced listeners show less intersubjective variability and therefore seem to be more stimulus driven. This expands the argument made in connection with Experiment 2, that listening strategies are an important component to explain phase alignment and degree of synchonization in tapping to quasi-periodic acoustic stimuli.

## General Discussion

The current study investigated motor responses and pulse experience, as manifest in response timing, to North Indian raga performances with three experiments. We used real music, excerpts from musical performances, as stimuli because the artificial stimuli frequently used in pulse and tapping research scarcely resemble examples of performed music, and conclusions drawn from such research have only limited and often questionable significance in explaining actual musical behavior.

In all three experiments participants responded to the stimuli with relatively steady and regular motor sequences, and participants expressed a regular pulse for both alap and jor/jhala, despite considerable differences in temporal regularities of these sections. While response rates to alap seem to be related to subjects’ internal periodicity, with little inter-subject agreement and responses only showing intermittent synchronization with the stimuli, response rates for the jor and jhala are shaped by the mean event rate of the stimuli, with considerable inter-subject agreement and a significant overall synchronization with the stimulus events.

**Fig 16:**
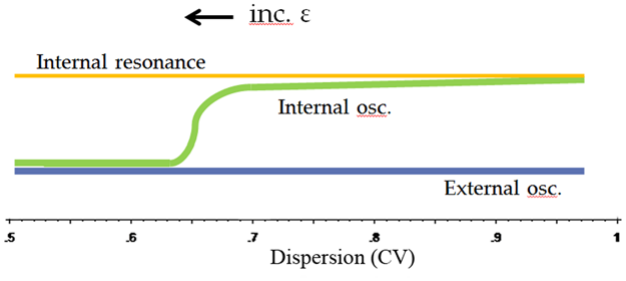
Entrainment model for non-isochronous auditory stimuli. A subject with internal periodicity (yellow) listens to an external acoustical event sequence (blue). As long as the dispersion of the event sequence is large, motor responses are determined by the subject’s internal resonance frequency and slightly modified by interactions with the external signal. When the periodicity of the external event sequence becomes more regular, and its CV is reduced to ca. 0.65 the interaction between internal and external oscillators increases beyond a critical value, at which point the periodicity of the motor responses becomes attracted to that of the external signal and adjusts to it.

Based on the results of these experiments we set out below a model of motor responses to irregular and quasi-regular musical stimuli that covers the processes analysed in the current study (see Fig 16). Our starting point is a central feature of sensori-motor synchronization, the internal or subjective periodicity, which links temporal aspects of both action- and perception- related processes. It is related to oscillatory neural mechanisms involved in both the preferred response period for repetitive motor actions and locomotion [34] and the tonic EEG synchronization response to periodic acoustic stimuli [33]. The internal periodicity synchronizes perceptual processing and action sequences by structuring perceptual intervals (attentional cycles) in accord with the resonance frequency of the motor system, and allows optimization of motor and perceptual performance [36]. Analyses of long-term movement spectra and EEG coherence patterns show that they comprise a dominant mode as well as a series of harmonically related minor modes. Experimental, analytical, and theoretical studies [37]–[38], [25], [34], [33] indicate a 2Hz resonance maximum for the dominant mode, with individual maxima varying between ca. 1.5 and 2.4 Hz (as indicated by EEG coherence measures; [39]).

Without external stimuli, subjects’ spontaneous motor activities (spontaneous tapping) appear to be determined by this internal periodicity, i.e. subjects tap in one of the response modes. The ‘multimodal’ structure of the internal periodicity is readily observable in frequency histograms of spontaneous motor responses (e.g. [24]–[25]; see also spontaneous responses of our Experiment 3).

The presence of an external stimulus series leads to modifications of the motor responses. With irregular stimuli such as the alap sections of the current study, responses are drawn towards modes with longer periods and inter-subject differences increase. Within the framework of the theory of dynamic attending [7], [29] these changes can be explained as reflecting subjects’ attention being drawn towards salient sound events, the timing of which may be more compatible with one mode than another, leading to adjustments of response modes and response timing, as indicated by the intermittent synchronization. Comparable effects of external stimulus sequences on repetitive motor tasks have also been reported for different experimental contexts, and are sometimes described as ‘unintentional entrainment’ [40]–[41]. Characteristic for this situation is that the individual response rates seem to be dominantly shaped by subjects’ internal periodicity.

This picture changes when temporal regularities are greater, as in the jor and jhala sections of our experiments. There is a high degree of inter-subject agreement on response rates, all response rates are now related to the average period of the stimulus event series, and responses show significant overall phase synchronization with the event sequences. That means, in the presence of the increased temporal regularity of the jor/jhala events the internal periodicities of the participants seem to adjust to rates that permit alignment with regularities of the stimuli. During the transition from alap to jor one observes small adjustments, when the alap response rate is close to one compatible with the jor, as well as larger jumps. This indicates that with the onset of the jor sections not only do adjustments of internal periodicities occur but there may also be changes in response mode. Sudden changes of response mode are also found within the jor or jhala sections but not during the alap. However, these changes of response mode, initiated when specific event features elicit attentional shifts, are only observed in a few participants, and it remains to be shown whether they are linked to specific musical experiences and/or strategies.

The data of the present study seem to suggest that the change in response characteristics from alap to jor (increased phase synchronization, inter-subject agreement) occurs in a rather sudden, rapid fashion once the temporal regularity reaches a certain degree. As we have mentioned above, some of our results indicated that the dispersion measures (CV / MAD) used here do not fully reflect the temporal regularity of our stimuli because they do not account for any recurrence of temporal patterns. However, as dispersion and recurrence are not independent characterizations of relevant stimulus aspects, we suspect that a future inclusion of suitable quantitative recurrence measures may possibly slightly alter, but not drastically change the observed relationship. We therefore suggest that the observed relationship can be described by an ‘entrainment function’, which in our musical context, has the following characteristics: adaptable oscillators at the base of the internal periodicities interact with fixed external event series; this interaction can lead to intermittent synchronization and slight modification of the response periodicities; with decreasing irregularity of the external signal, a change from intermittent to continuous synchronization and entrainment by external periodicities occurs rather suddenly (Fig 16). This does not seem to be an uncommon state change as comparable functions with sharp transitions have been identified in theoretical and experimental studies on entrainment in chemo-physiological and behavioral systems [42]–[43].

On the basis of the current study we propose that pulse should be understood as the experience of one’s internal periodicity. As such it is not necessarily linked to temporally regular, structured sensory input streams. It arises spontaneously through the performance of repetitive motor actions (spontaneous tempo), or even by exposure to event sequences with rather irregular temporal structures (alap sections). What changes when a sensory input reaches a certain degree of regularity is the emergence of increased interaction and alignment between external events and internal periodicity. The synchrony between the two may modify the ‘feel’ of the pulse, but most importantly it transforms the subjective experience of pulse into a shared one: subjects experience internal periodicities that are either identical or harmonically related. This may be one of the most significant inter-personal effects of the temporal regularity of music.

The concept of pulse proposed here is supported by the view of many Indian musicians that ‘there is a pulse’ in alap [11], and it offers an interesting reinterpretation of the dominant view, mentioned in the introduction, that alap is not supposed to have a pulse: it can be seen as an aesthetic imperative to overcome, or at least to hide, the natural and hardly avoidable tendency to perform repetitive motor actions in a temporally regular manner. One way of approaching this aesthetic goal, as it emerges from the analysis of the different alaps used in the current study, is to relegate regularities as much as possible to less salient feature levels, e.g. to chikari events instead of the main melody events. Then the regularity of the chikari events could be seen as a reflection of the performer’s subjective periodicity that, however, is not easily accessible to the listener because it is broken down, blurred, and hidden by the greater salience of the interspersed melodic events; after all, the main purpose of the alap is the melodic exposition of a rag.

The principle of musical development from irregular to more regular timing and from slow to fast is common to many Indian classical music genres, but there are also differences in the way this is realized—between genres, between vocal and instrumental performance, and between different instruments. We would anticipate similar results with another north Indian plucked instrument such as the sitar, for example, while responses to alap performed using the voice or bowed or blown instruments may show some differences and would be worth investigating for this reason. Similarly, cognate traditions over a wider geographical area (such as the Arabo-Turkish taksim) would be worth investigating: we would hypothesize that a similar entrainment function would be engaged, although differences in the stimulus structure may nonetheless produce differences in the particular response patterns.

## Supporting Information

S1 Fig Phase histograms of response phases for alap and jor sections from three selected participants. s12 (O group) and s9 (K group) show significant phase alignment for the alap and the jor, whereas s2 (K group) only shows synchronization for the jor.
(TIF)

S2 Fig Phase plots of responses to the slow and fast version of the stimulus from three participants. One shows no phase alignment (s2, the only subject whose response ratio corresponds to the transformation ratio of the stimuli); one shows phase alignment in both versions (s8); and one shows alignment in the fast but not in the slow version (s14).
(TIF)

S3 Fig Rate changes in spontaneous clapping of four subjects. Order of tap sequences (n) plotted against the clap interval duration (s).
(TIF)

S1 Table Individual results for experiment 3. (DOCX)
