# Supplementary figures and images for: Pulse and Entrainment to Non-Isochronous Auditory Stimuli: The Case of North Indian Alap
Source: PLoS One. 2015 Apr 7;10(4):e0123247. doi: 10.1371/journal.pone.0123247 (PMC4388701; doi:10.1371/journal.pone.0123247)

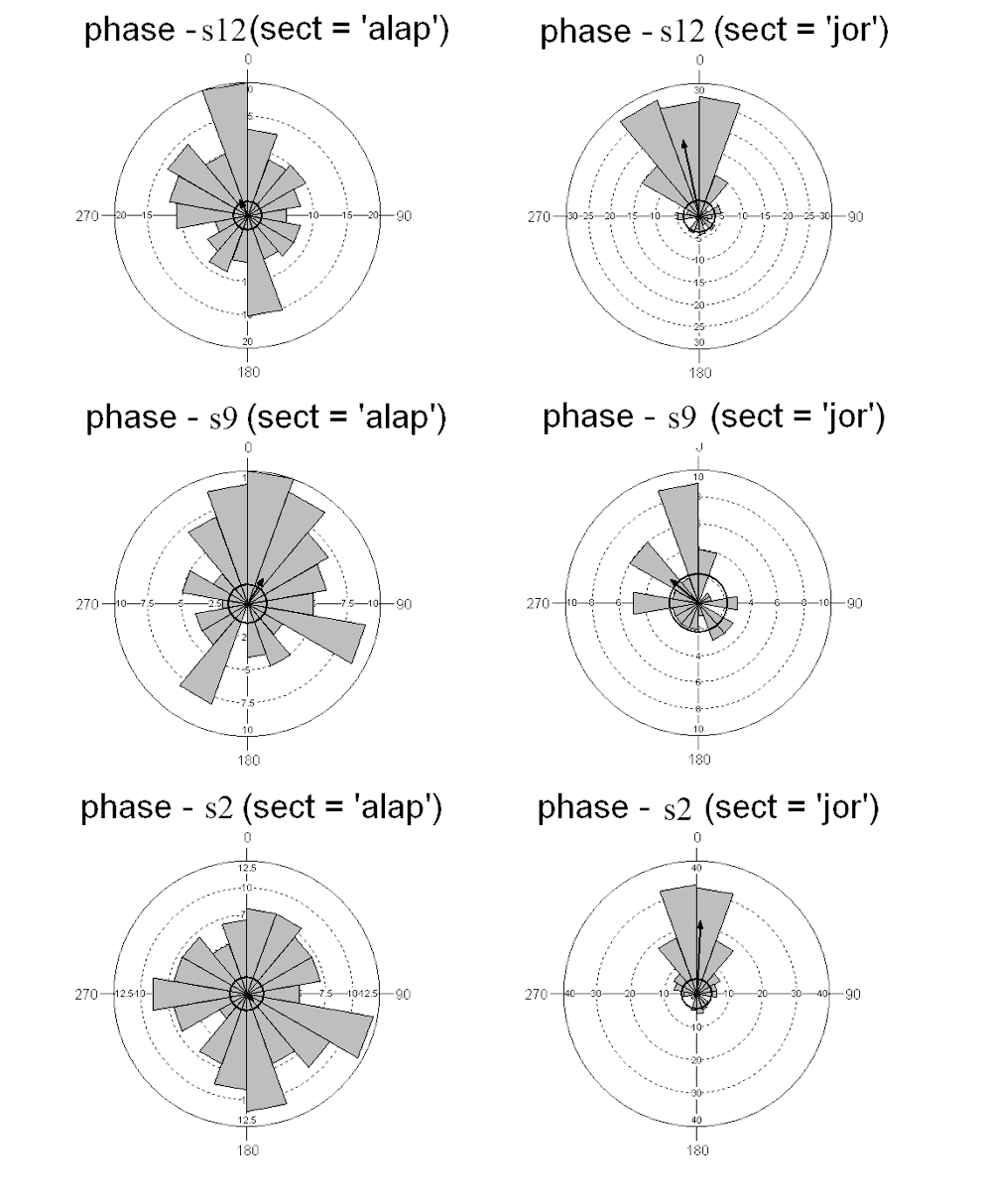

Supplement: S1 Fig — s12 (O group) and s9 (K group) show significant phase alignment for the alap and the jor, whereas s2 (K group) only shows synchronization for the jor. (TIF) [file pone.0123247.s001.tif]

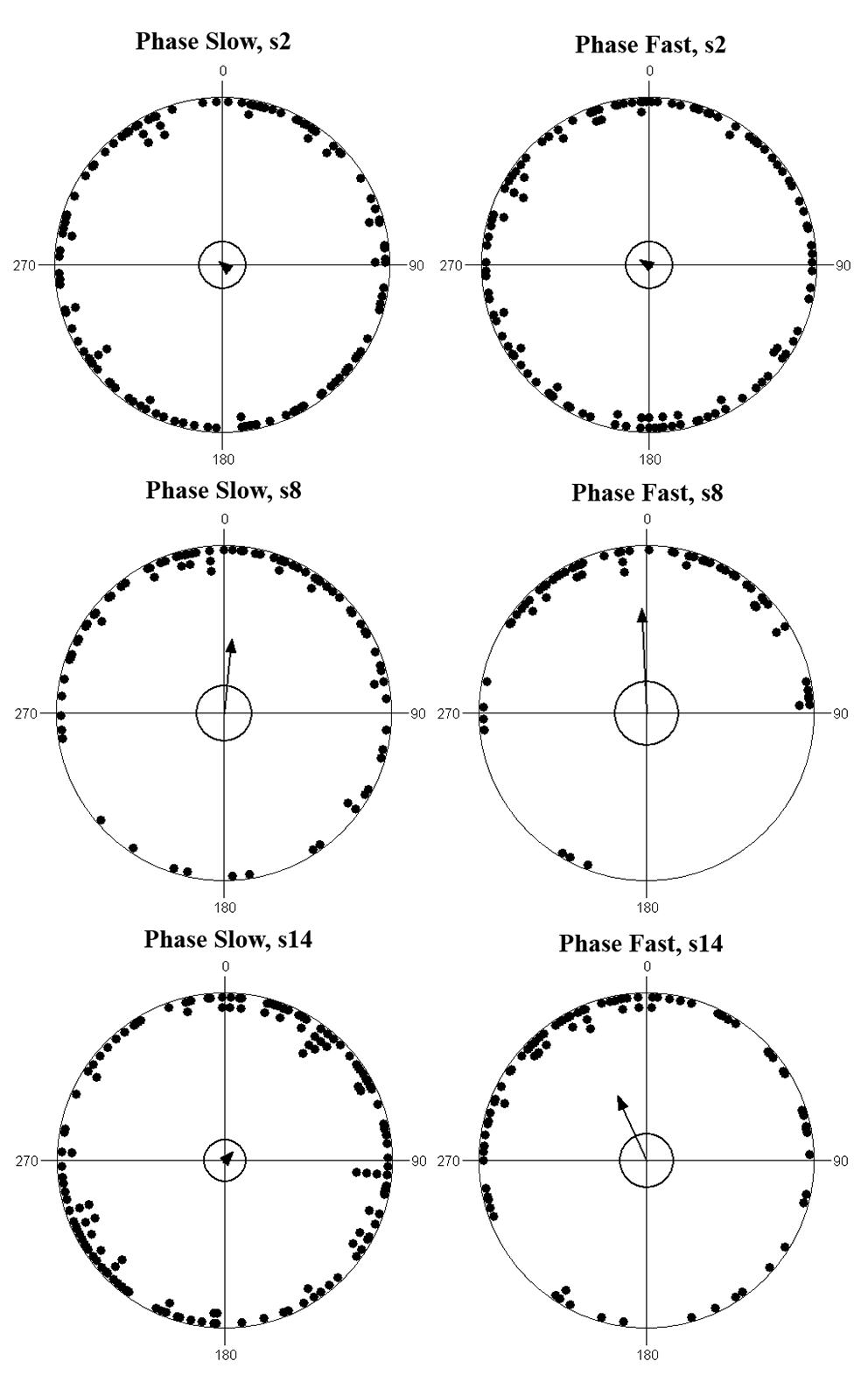

Supplement: S2 Fig — One shows no phase alignment (s2, the only subject whose response ratio corresponds to the transformation ratio of the stimuli); one shows phase alignment in both versions (s8); and one shows alignment in the fast but not in the slow version (s14). (TIF) [file pone.0123247.s002.tif]

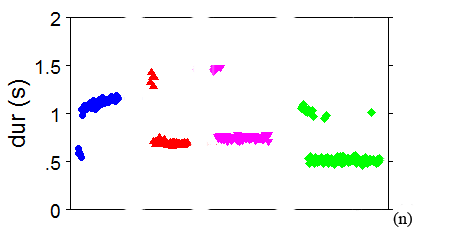

Supplement: S3 Fig — Order of tap sequences (n) plotted against the clap interval duration (s). (TIF) [file pone.0123247.s003.tif]
