# Supplementary material for: Pulse and Entrainment to Non-Isochronous Auditory Stimuli: The Case of North Indian Alap
Source: PLoS One. 2015 Apr 7;10(4):e0123247. doi: 10.1371/journal.pone.0123247 (PMC4388701; doi:10.1371/journal.pone.0123247)
Supplement: S1 Table — (DOCX) [file pone.0123247.s004.docx]

Table A: Individual results for Gaud Sarang

|  | Gaud Sarang | | | | | | | |
| --- | --- | --- | --- | --- | --- | --- | --- | --- |
|  | Alap 1 | | Alap 2 | | Jor | | Jhalla | |
| ID | µ/r | V/p(V) | µ/r | V/p(V) | µ/r | V/p(V) | µ/r | V/p(V) |
| S1 | NR | NR | 213.5/0.30 | 1.3/>0.15 | **33.9/0.53** | **6.52/<0.01** | **69.9/0.39** | **4.21/<0.01** |
| S2 | **344.6/0.20** | **1.78/<0.05** | 31.2/0.03 | 0.87/>0.15 | **33.4/0.56** | **4.93/<0.01** | **61.0/0.19** | **2.27/<0.01** |
| S3 | 219.3/0.18 | 1.51/>0.15 | **267.9/0.22** | **1.86/<0.05** | **332.6/0.53** | **6.18/<0,01** | **236.4/0.07** | **1.86/<0.05** |
| S4 | 310.7/0.17 | 1.26/>0.15 | 34.2/0.19 | 1.62/>0.10 | **12.1/0.55** | **5.96/<0.01** | **350.3/0.30** | **3.51/<0.01** |
| S5 | 180.8/0.09 | 0.09/>0.15 | 286.0/0.10 | 1.02/>0.15 | **187.7/0.15** | **2.28/<0.01** | **241.6/0.13** | **2.32/<0.01** |
| S6 | 10.2/0.10 | 1.55/>0.10 | 37.8/0.16 | 1.63/0.06 | **16.9/0.61** | **7.67/<0.01** | **10.3/0.30** | **3.97/<0.01** |
| S7 | **80.7/0.39** | **2.08/<0.01** | 63.2/0.18 | 1.30/>0.15 | 123.3/0.14 | 1.56/>0.10 | 160.7/0.08 | 0.97/>0.15 |
| S8 | **345.0/0.26** | **2.93/<0.01** | 20.4/0.12 | 1.60/>0.10 | **33.2/0.58** | **6.26/<0.01** | **357.8/0.20** | **2.46/<0.01** |
| S9 | **338.6/0.28** | **1.54/<0.01** | **27.1/0.34** | **1.86/<0.05** | **59.2/0.46** | **5.42/<0.01** | **64.1/0.28** | **3.17/<0.01** |
| S10 | NR | NR | 216.3/0.18 | 1.69/0.06 | **146.9/0.34** | **4.19/<0.01** | **248.4/0.19** | **2.48/<0.01** |
| S11 | 307.8/0.11 | 1.21/>0.15 | 338.4/0.16 | 1.71/0.06 | 217.8/0.04 | 1.21/>0.15 | **316.9/0.19** | **2.22/<0.01** |
| S12 | 9.9/0.29 | 1.20/>0.15 | 53.5/0.09 | 0.88/>0.15 | **7.46/0.58** | **7.22/<0.01** | **358.3/0.30** | **3.82/<0.01** |
| S13 | **47.0/0.33** | **1.76/<0.05** | 357.7/0.13 | 1.20/>0.15 | **329.7/0.39** | **4.63/<0.01** | **331.9/0.18** | **2.98/<0.01** |

ID = subject ID; µ = mean vector (degree); r = length of mean vector; V = Kuiper test V; p(V) = probability for V. Responses significant at α=0.05 are marked bold.

Table B: Individual results for Jaijaiwanti

|  | Jaijaiwanti | | | | | | | |
| --- | --- | --- | --- | --- | --- | --- | --- | --- |
|  | Alap 1 | | Alap 2 | | Jor | | Jhalla | |
| ID | µ/r | V/p(V) | µ/r | V/p(V) | µ/r | V/p(V) | µ/r | V/p(V) |
| S1 | NR | NR | NR | NR | **312.3/0.55** | **5.55/<0.01** | 223.1/0.06 | 1.40/>0.15 |
| S2 | **29.3/0.14** | **2.25/<0.01** | 346.4/0.02 | 1.00/>0.15 | **34.4/0.51** | **6.41/<0.01** | **43.9/0.43** | **4.42/<0.01** |
| S3 | 347.3/0.07 | 1.38/>0.15 | 98.5/0.03 | 0.92/>0.15 | **11.3/0.46** | **7.57/<0.01** | **356.1/0.23** | **3.32/<0.01** |
| S4 | 301.2/0.07 | 1.20/>0.15 | 287.8/0.10 | 1.32/>0.15 | **21.9/0.41** | **5.35/<0.01** | 58.2/0.05 | 1.42/>0.15 |
| S5 | 68.8/0.04 | 0.87/>0.15 | 321.6/0.13 | 1.47/>0.15 | **244.3/0.11** | **2.20/<0.01** | 31.5/0.09 | 1.47/>0.15 |
| S6 | **335.2/0.16** | **2.00/<0.05** | 354.7/0.14 | 1.55/0.06 | **327.5/0.49** | **8.24/<0.01** | **356.0/0.23** | **3.07/<0.01** |
| S7 | 183.7/0.11 | 1.16/>0.15 | 109.9/0.10 | 1.24/>0.15 | **255.4/0.10** | **1.80/<0.05** | **277.6/0.14** | **1.89/<0.05** |
| S8 | 258.2/0.07 | 1.22/>0.15 | 176.6/0.06 | 1.29/>0.15 | 286.1/0.06 | 1.42/>0.15 | 273.9/0.04 | 1.10/>0.15 |
| S9 | **3.9/0.19** | **1.75/<0.05** | 159.6/0.12 | 1.38/>0.15 | 318.9/0.48 | 5.32/<0.01 | **338.9/0.37** | **3.94/<0.01** |
| S10 | **357.5/0.14** | **2.08/<0.01** | 73.3/0.06 | 1.17/>0.15 | 356.7/0.51 | 8.80/<0.01 | **359.2/0.35** | **4.56/<0.01** |
| S11 | **349.7/0.17** | **2.20/<0.01** | 356.2/0.07 | 1.46/>0.15 | 356.8/0.07 | 1.77/<0.05 | **77.6/0.11** | **2.28/<0.01** |
| S12 | 340.1/0.12 | 1.51/>0.15 | 21.6/0.10 | 1.46/>0.15 | 6.7/0.56 | 9.28/<0.01 | **7.7/0.37** | **5.13/<0.01** |
| S13 | 154.2/0.30 | 1.47/>0.15 | 311.0/0.10 | 1.16/>0.15 | 299.7/0.33 | 5.23/<0.01 | **355.6/0.30** | **4.27/<0.01** |

ID = subject ID; µ = mean vector (degree); r = length of mean vector; V = Kuiper test V; p(V) = probability for V. Responses significant at α=0.05 are marked bold.

Table C: Individual results for Kafi Kanada

|  | Kafi Kanada | | | | | |
| --- | --- | --- | --- | --- | --- | --- |
|  | Alap 1 | | Alap 2 | | Alap 3 | |
| ID | µ/r | V/p(V) | µ/r | V/p(V) | µ/r | V/p(V) |
| S1 | NR | NR | NR | NR | NR | NR |
| S2 | 10.3/0.06 | 1.36/>0.15 | 108.5/0.11 | 1.48/>0.15 | 154.5/0.07 | 0.94/>0.15 |
| S3 | 100.1/0.06 | 1.13/>0.15 | 268.1/0.05 | 1.06/>0.15 | **198.1/0.15** | **1.88/<0.05** |
| S4 | 351.2/0.05 | 1.09/>0.15 | 27.6/0.14 | 1.61/0.06 | 0.2/0.08 | 1.33/>0.15 |
| S5 | 254.9/0.04 | 1.05/>0.15 | 9.3/0.10 | 1.31/>0.15 | 187.9/0.03 | 1.04/>0.15 |
| S6 | 330.3/0.06 | 1.45/>0.15 | 56.6/0.05 | 1.02/>0.15 | 309.4/0.03 | 1.04/>0.15 |
| S7 | **43.5/0.26** | **1.59/<0.05** | 77.93/0.03 | 1.09/>0.15 | 27.5/0.03 | 1.00/>0.15 |
| S8 | 229.2/0.05 | 1.45/>0.15 | 50.90/0.10 | 1.45/>0.15 | 354.4/0.08 | 1.48/>0.15 |
| S9 | **91.7/0.74** | **3.95/<0.01** | **86.4/0.24** | **1.70/<0.05** | **110.6/0.28** | **2.39/<0.01** |
| S10 | 348.6/0.09 | 1.59/>0.10 | 181.6/0.06 | 1.18/>0.15 | 277.3/0.14 | 1.62/>0.06 |
| S11 | 252.7/0.02 | 0.88/>0.15 | **52.0/0.13** | **1.95/<0.05** | 344.5/0.06 | 1.18/>0.15 |
| S12 | 216.0/0.07 | 1.41/>0.15 | 6.6/0.07 | 1.13/>0.15 | 2.8/0.11 | 1.49/>0.15 |
| S13 | NR | NR | 210.2/0.07 | 0.99/>0.15 | 86.6/0.51 | 1.19/>0.15 |

ID = subject ID; µ = mean vector (degree); r = length of mean vector; V = Kuiper test V; p(V) = probability for V. Responses significant at α=0.05 are marked bold.
